# Polysaccharides from the leaves of *Polygonatum sibiricum* Red. regulate the gut microbiota and affect the production of short-chain fatty acids in mice

**DOI:** 10.1186/s13568-022-01376-z

**Published:** 2022-03-21

**Authors:** Yu Luo, Qi Fang, Yong Lai, Hui Lei, Dan Zhang, Hong Niu, Rui Wang, Can Song

**Affiliations:** School of Pharmacy, Southwest Medical University, Luzhou, 646000 Sichuan China

**Keywords:** *Polygonatum sibiricum*, Polysaccharides, Gut microbiota, High-throughput sequencing, Short-chain fatty acids

## Abstract

Polysaccharides from the rhizome of *Polygonatum sibiricum* display a variety of biological activities, including the regulation of intestinal microbiota, but the polysaccharides from the leaves of *P. sibiricum* have not been studied extensively. Here, we extracted crude polysaccharides from the leaves of *P. sibiricum* and further separated and purified them to study the effects of *P. sibiricum* polysaccharides (PsPs) on intestinal microbes and short-chain fatty acids (SCFAs). The PsPs had a total sugar content of 97.48% and a monosaccharide composition comprising mannose, rhamnose, galacturonic acid, glucose, xylose, and arabinose, with molar ratios of 6.6:15.4:4.5:8.8:40.7:24, respectively. The effects of PsPs on intestinal microflora in mice were also studied, with 16S sequencing results showing an increase in the relative abundance of *Firmicutes* and a decrease in *Bacteroidetes* at the phylum level. The abundance of *Lactobacillus* increased, while those of *Lachnospiraceae* and *Bacteroides* reduced (at the genus level) by PsPs treatment. The composition of microbes changed. Levels of SCFAs in the PsPs group were significantly increased compared with control mice, including acetic acid, propionic acid, and butyric acid. These results suggest that PsPs can act as prebiotics, regulating the intestinal tract probiotics.

## Introduction

The traditional Chinese medicine *Polygonatum Rhizoma* includes dried rhizomes of *Polygonatum kingianum, Polygonatum sibiricum,* or *Polygonatum cyrtonema*. *Polygonatum* plants, which include more than 40 species, are widely distributed throughout the temperate northern hemisphere (Zhao et al. 2018). The plant invigorates the Qi (Traditional Chinese medicine believes that Qi is one of the basic substances that constitute the human body and maintain its vital activities) and spleen, moistening the lungs and the kidneys, and is used in clinical medicine (Li et al. 2021). The medicinal value of *Polygonatum* is derived from its chemical components, including polysaccharides (Liu et al. 2007), steroidal saponins (Mi-Jeong et al. 2006), alkaloids (Sun et al. 2005), flavonoids, and phytosterols (Juan et al. 2016). Accumulating evidence suggests that high-molar-mass polysaccharides are the major components of herbal medicines (Jia et al. 2019).

Plant polysaccharides, especially some Chinese herbal polysaccharides, have a wide range of pharmacological properties, including unique effects as antioxidants (Wang et al. 2017; Debnath et al. 2013), antidiabetics (Shu et al. 2012), antineoplastics (Senthilkumar et al. 2013; Sajadimajd et al. 2019), anti-inflammatories (Jun et al. 2015), and anti-atherosclerosis (Zeng et al. 2011) and immunocompetence agents (Kim et al. 2019), as well as having bone-protective effects (Lv et al. 2016). Chinese herbal polysaccharides are basically non-toxic to the body; as such, they have attracted much interest from researchers (Zhao et al. 2018) and are gradually being developed into medical products with clinical value (Xiao et al. 2018), such as the polysaccharides from *Astragalus* (Lv et al. 2017), *Ginseng* (Shalaby et al. 2018), and *Poria cocos* (Wu et al. 2018). *Polygonatum* polysaccharides can promote the growth of probiotics by regulating the structure and composition of intestinal microorganisms (Wang et al. 2018; Yang et al. 2021). At present, *Polygonatum* polysaccharides are mainly extracted from the rhizome of plants. Previous studies have shown that for every ton of rhizomes collected, about 400 kg of stems and leaves are produced (Yang et al. 2020); however, these are usually discarded, making the whole plant underutilized. Therefore, multi-angle and deep utilization of *Polygonatum* can effectively improve its comprehensive value. In particular, the potential effects, structural characteristics, and biological activity of polysaccharides extracted from *P. sibiricum* leaves have not been examined. Therefore, in this study we extracted, separated, and purified crude polysaccharides from the leaves of *P. sibiricum* Red. We then investigated the effects of purified *P. sibiricum* polysaccharides (PsPs) on intestinal microorganisms and short-chain fatty acids (SCFAs) in mice. Our results suggest that PsPs have the potential to regulate intestinal flora and protect intestinal health as prebiotics.

## Materials and methods

### Raw materials and chemical reagents

Mature healthy *P. sibiricum* Red. leaves were collected from the *Polygonatum* planting (no dead leaves and young leaves) base in Xuyong County, Luzhou City, China. Standard monosaccharides (mannose, rhamnose, and glucuronic acid) were purchased from Shanghai yuanye Bio-Technology Company, China. Standard SCFAs (acetic acid, butyric acid) were purchased from SIGMA Company, China. All other reagents provided by the laboratory were analytical grade.

### Preparation of PsPs

Traditional water extraction and alcohol precipitation methods were used to extract crude polysaccharides (Li et al. 2020). First, the *Polygonatum* leaves were placed in a beaker with twice the volume of water and then heated in a water bath at 90 °C for 3 h and extracted three times. The extracts were then concentrated to 2 L, and 1.5-times the volume of anhydrous ethanol was added to a final concentration of 60% ethanol. After storing at room temperature for 24 h, the concentrate was filtered to obtain the crude polysaccharides. Finally, the extracted crude polysaccharides were dissolved in 1 L of distilled water and decolorized with a macroporous resin. Subsequently, the crude polysaccharide solution was purified with DEAE cellulose and dialyzed for 72 h in a dialysis bag (500–1 KD. After dialysis, the purified polysaccharides were concentrated and freeze-dried.

### Characterization of PsPs

The phenol-sulfate method with a glucose standard as the control was used to determine total sugar content according to Herbert D (1971). Absorbance was measured using a UV–visible spectrophotometer at 490 nm, and the total sugar content was calculated from a standard curve.

The monosaccharide composition of the polysaccharides was determined using high performance liquid chromatography (HPLC), as described by Dai et al. (2010). The operational steps were as follows: (1) Derivatization of single and mixed marks: standard xylose, mannose, rhamnose, arabinose, galacturonic acid, glucose, glucuronic acid, and galacturonic acid were prepared at 0.5 mg/mL in a mixed standard solution. The PMP derivatization reaction was performed at 70 °C for 100 min, before cooling the solution at room temperature for 5–10 min; thereafter, 2 mL of 0.3 M HCl was added to neutralize added NaOH. After thorough shaking, an equal volume of chloroform was added for extraction. The water phase obtained was filtered through a 0.22 mm membrane and determined by liquid chromatography. (2) Hydrolysis and derivatization of the sample: an appropriate amount of dried polysaccharide sample was weighed and placed in a capped reaction tube with 4–6 mL of 2 M TFA, and reacted with N_2_ for 120–150 min at 121 °C. The sample was cooled to room temperature, after which 0.2 mL methanol was added and the pressure was reduced. The concentrate was then dried. The procedure was repeated several times until the TFA was completely removed. After adding distilled water (0.5 mL) to dissolve the hydrolyzed sample, PMP derivatization followed the same procedure, including filtering through a 0.22 mm filter membrane, and analysis by liquid chromatography using an Agilent 1100 HPLC system. The chromatographic column used was C18 (250 nm × 4.6 nm). The injection volume was 20 ml, and an 83:17 volume ratio of phosphoric acid buffer and acetylene mixture was used as the mobile phase. The flow rate was 1 ml/min. An SPD-15C UV–visible detector with a detection wavelength of 254 nm was used and the column temperature was 30 °C.

### Animal experiments

Twelve male Kunming mice (BW 20 ± 2 g) were purchased from the Experimental Animal Center of Southwest Medical University. The mice were kept in a room with 60 ± 10% relative humidity, a temperature of 23 ± 2 ℃, and a 12 h light/dark cycle, with food and water. All animal care and procedures were performed in accordance with the approved guidelines and regulations of the Animal Care and Use Committee of the SWMU. After 7 days of captive feeding, the mice were randomly divided into two groups of six mice: (1) control group: mice were fed once a day and were provided with the same volume of distilled water as the experimental group; and (2) PsPs group: mice were fed PsPs (200 mg/kg) once a day. The only variable was the addition of PsPs; thus, the background was clear and the repeatability was good. The activity and mental state of the mice were observed, and their body weights recorded. Mouse feces were collected at the end of the experiment, and the samples were stored at −80 °C for further comprehensive analysis.

### Short-chain fatty acid (SCFA) analysis

The contents of SCFAs (acetic acid, propionic acid, butyric acid, iso-butyric acid, valeric acid, isovaleric acid, hexanoic acid, and iso-hexanoic acid) were determined by GC–MS.

Stool sample (25 mg) was placed in a 2 ml grinding tube, and 500 μL water (containing 0.5% phosphoric acid) was added. N-butanol solvent (containing the internal standard 2-ethylbutyric acid at 10 μg/mL) was added for extraction using low-temperature ultrasound for 10 min. The samples were then centrifuged at 13,000 *g* at 4 °C for 5 min, and then filtered through a 0.22-μm filter membrane. An Agilent Technologies Inc. (CA, USA) 8890B-5977B GC/MSD GC/MSD was used as the analysis instrument. Chromatographic conditions were as follows: HP FFAP capillary column (30 m × 0.25 mm × 0.25 μm; Agilent J&W Scientific company, Folsom, CA, USA), high purity helium carrying gas, flow rate of 1.0 mL/min, and inlet temperature of 260 °C. The injection volume was 1 μL, applied as a split injection at a split ratio of 10:1 and a solvent delay of 2.5 min. The initial temperature of the column chamber was 80 °C, and then the temperature was programmed to cycles of 120 °C at 40 °C/min, 200 °C at 10 °C /min, and then maintained at 230 °C for 3 min.

### 16S ribosomal RNA (rRNA) gene and bioinformatics analysis

The V3–V4 16S rRNA region, a highly variable region of the gene, was amplified according to Guo et al. (2020). High-throughput sequencing of the products was carried out by Majorbio Company (Shanghai, China). QIIME (version 1.9.1) was used for demultiplexing and quality filtering of all sequences before data analysis. Some reads were discarded, such as reads that could not be assembled. For 16S rDNA, sequences with 100% similarity were classified as amplicon sequence variant (ASVs). Chimeric sequences, which were identified and using UCHIME program, were deleted. The Wilcoxon rank sum-test was used to analyze differences among the experimental group and the control group at different levels (phylum level and genus level). Principal Coordinate Analysis (PCoA) was used to determine the component differences between the two groups at the phylum level, using the R package. Linear discriminant analysis effect size (LEfSe) was used to analyze bacterial composition differences. Correlation analysis was used to explore the interaction between intestinal flora and SCFAs. The correlation analysis identified significant correlations, strong correlations, positive correlations, and negative correlations between microbial communities and SCFAs. A correlation coefficient 0.6^3^ and P < 0.05 indicated a positive correlation.

### Statistical analysis

Data are expressed as the means ± standard deviations (SDs). GraphPad Prism version 7.04 was used to evaluate the significance differences between groups. Statistical significance was set at P < 0.05.

## Results

### Total sugar content and monosaccharide composition of PsPs

The total sugar content of the purified PsPs was 97.48% (Table 1). The purified PsPs were composed of mannose, rhamnose, galacturonic acid, glucose, xylose, and arabinose, with molar ratios of 6.6:15.4:4.5:8.8:40.7:24, respectively.

**Table1:** Total sugar content and monosaccharides of PsPs

	PsPs
Total sugar content (% W/W)	97.48
Monosaccharide (mol %)	
Man	6.6
Rha	15.4
Gala	4.5
Glc	8.8
Xyl	40.7
Ara	24

### Regulation of body weight by intragastric administration of PsPs

After intragastric administration of PsPs to mice for 3 weeks, their growth and mental condition remained normal, hair color was normal, and no abnormal deaths occurred. The t PsPs had no adverse effect on the growth of mice; during the experiment, the bodyweight of each group increased. By the end of the experiment, mice in the PsPs group had gained less body weight than those in the ConT group (Table 2).

**Table 2:** Effects of PsPs on body weight

Groups	Original weight (g)	The final weight (g)	Percentage of weight gain (%)
ConT	31.70 ± 1.58	37.56 ± 2.69	18.48
PsPs	30.81 ± 1.40	35.88 ± 2.97	16.45

### Gut microbiota composition

**Fig. 1:**
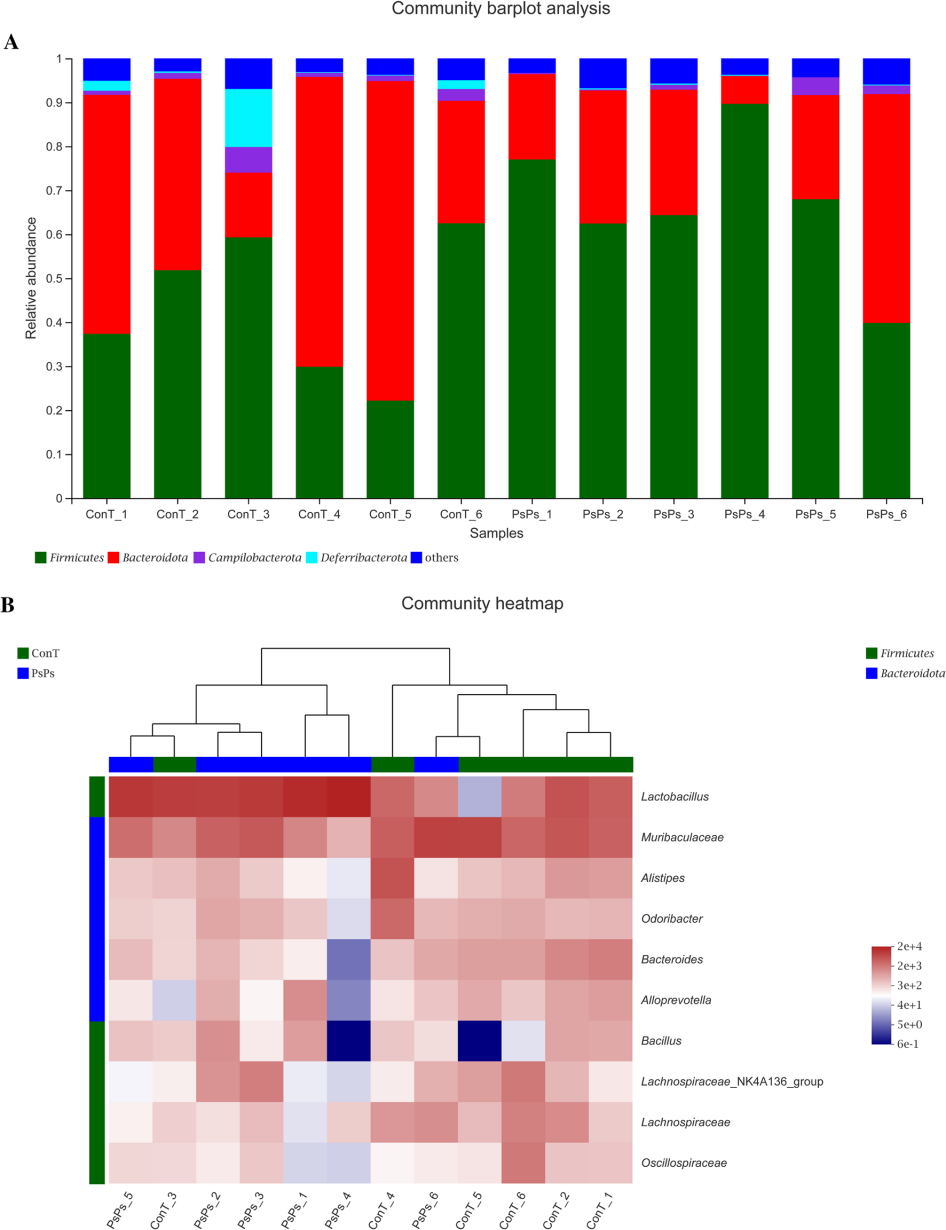
Regulation of polysaccharide on intestinal microflora composition in mice. **A** Relative abundance of intestinal flora at phylum level. **B** Classification heat map of intestinal flora at genus level

**Fig. 2:**
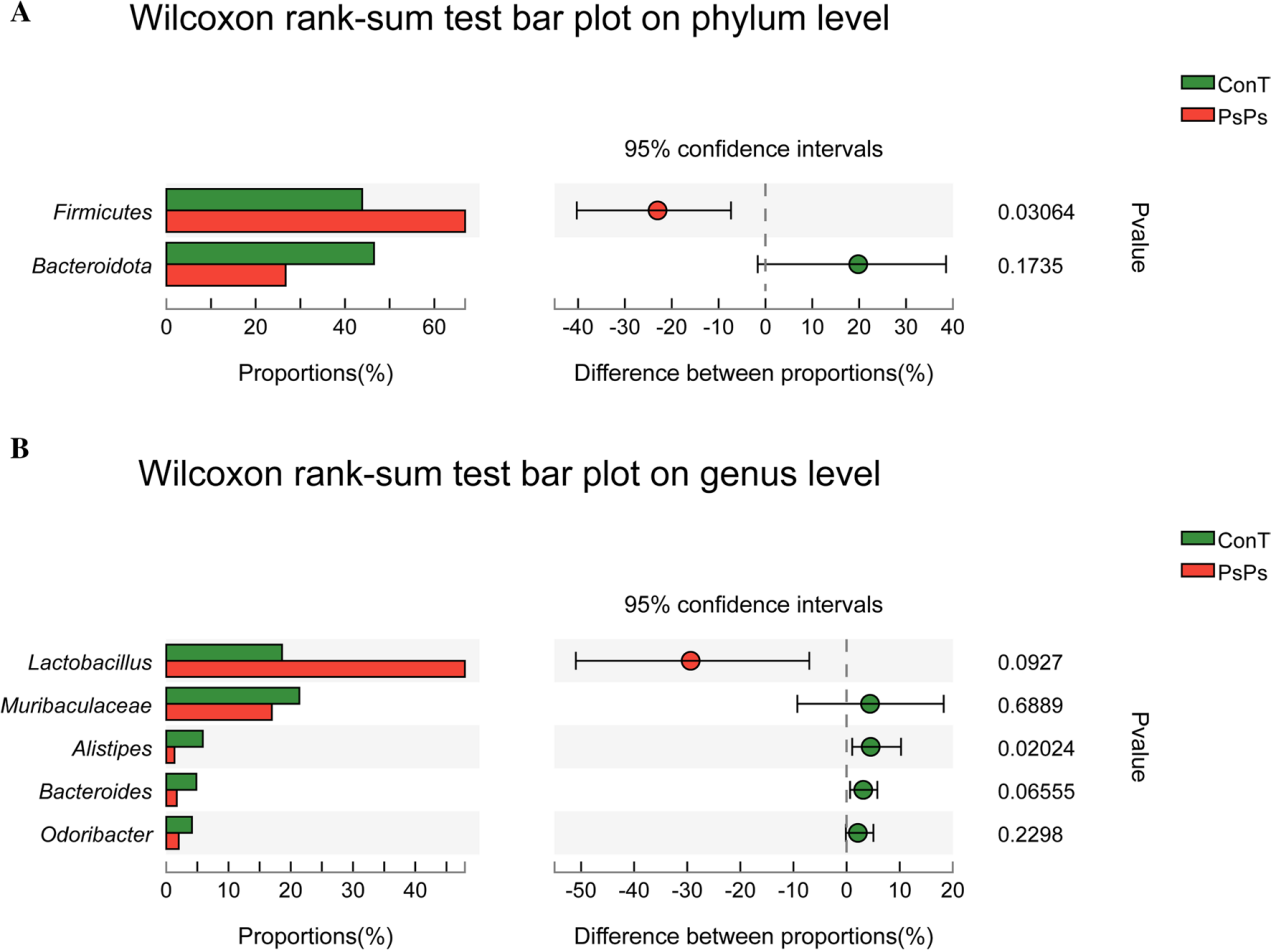
Wilcoxon rank sum test of gut microbiota. **A** The relative abundance of gut microbiota at the phylum levels. **B** The relative abundance of gut microbiota at the genus levels

Six samples were selected from the two groups of mice for high-throughput sequencing and 12 samples were collected. As shown in Fig. 1A, at the phylum level, the intestinal flora of mice in each group mainly consisted of *Firmicutes, Bacteroidetes, Campilobacterota,* and *Deferribacterota*. Compared with the control group, PsPs treatment resulted in an increase in the relative abundance of *Firmicutes,* but a decrease in the relative abundance of *Bacteroidetes* at the phylum level. Data can be reflected by a two-dimensional matrix or a table of heat map color changes, intuitively representing data values by color depth. At the genus level, as shown in Fig. 1B, there were three significant changes in the fecal flora representation of PsPs-treated mice compared with control mice, including increased *Lactobacillus* and decreased *Lachnospiraceae* and *Bacteroides*. The Wilcoxon rank-sum test bar plot was used to analyze the diversity of microbial composition at the phylum and genus level (Fig. 2). At the phylum level, the proportion of *Firmicutes* in the PsPs-treated group increased compared with the control group, while that of *Bacteroidetes* decreased (Fig. 2A). The results are consistent with those in Fig. 1. At the genus level (Fig. 2B), the proportion of *Lactobacillus* increased, but the percentage of *Muribaculaceae*, *Alistipes*, *Bacteroides*, and *Odoribacter* decreased.

### Intestinal flora structure

**Fig. 3:**
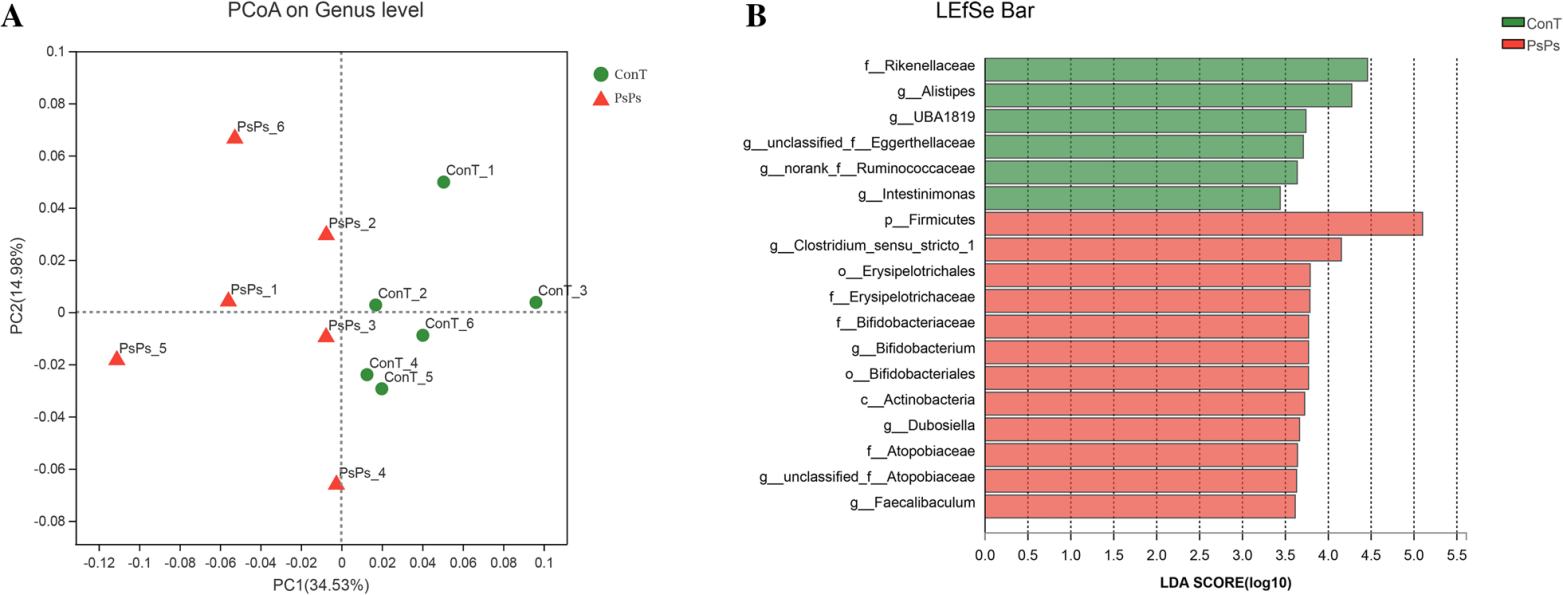
**A** PCoA analysis was performed based on weighted UniFrac distances. **B** The LDA scores of the PsPs group and the ConT group were obtained by LEfSe analysis

PCoA analysis was used to study the overall changes in the intestinal microbial community structure at the genus level (Fig. 3A). There were two principal components, and their contribution rate to the cumulative variance was 49.51%, indicating that the experimental group and the control group were significantly different in microbial community structure. Further, LEfSe was used to determine the bacterial community structure between the PsPs and conT groups (Fig. 3B). The results indicated that the genera *Alistipes* and *UBA1819*, and family *Rikenellaceae* in the ConT group; and phylum *Firmicutes*, genus *Clostridium *sensu stricto* 1*, and order *Erysipelotrichales* in the PsPs group were the main bacteria that caused the difference in the structure of intestinal flora between the two groups.

### Effect of PsPs on SCFAs production

**Fig. 4:**
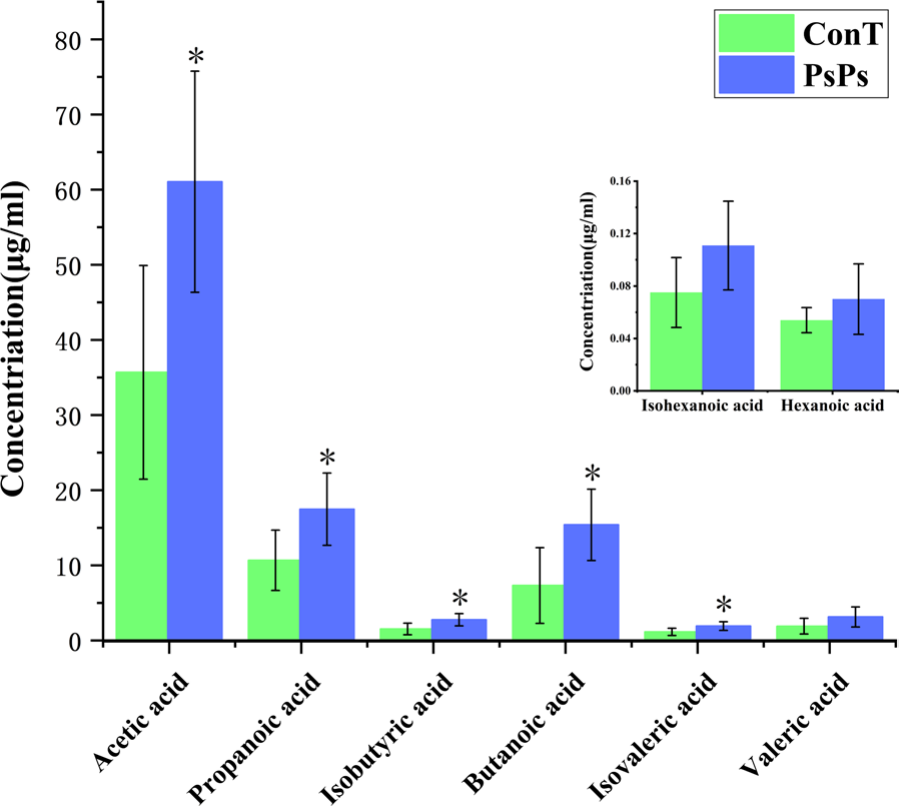
Changes of SCFAs concentration in feces of mice 3 weeks after PsPs intragastric administration. The Y-axis is the concentration of SCFAs in the samples determined by GC, in units of (ug/ mL), and all samples determined have the same weight

Many metabolites exist in the gut microbiota, including SCFAs, which are indispensable for host metabolism and immune protection. GC–MS analysis showed that the main SCFAs in feces were acetic acid, propionic acid, and butyric acid (Fig. 4). Compared with the ConT group, the concentrations of acetic acid, propionic acid, and butyric acid in the PsPs group were significantly increased (P < 0.05), while the concentrations of pentanoic acid, hexanoic acid, and isohexanoic acid did not significantly increase. Although the concentrations of iso-butyric and iso-valeric acids were relatively low in the PsPs group, they were considerably higher than those in the ConT group.

### Correlation

**Fig. 5:**
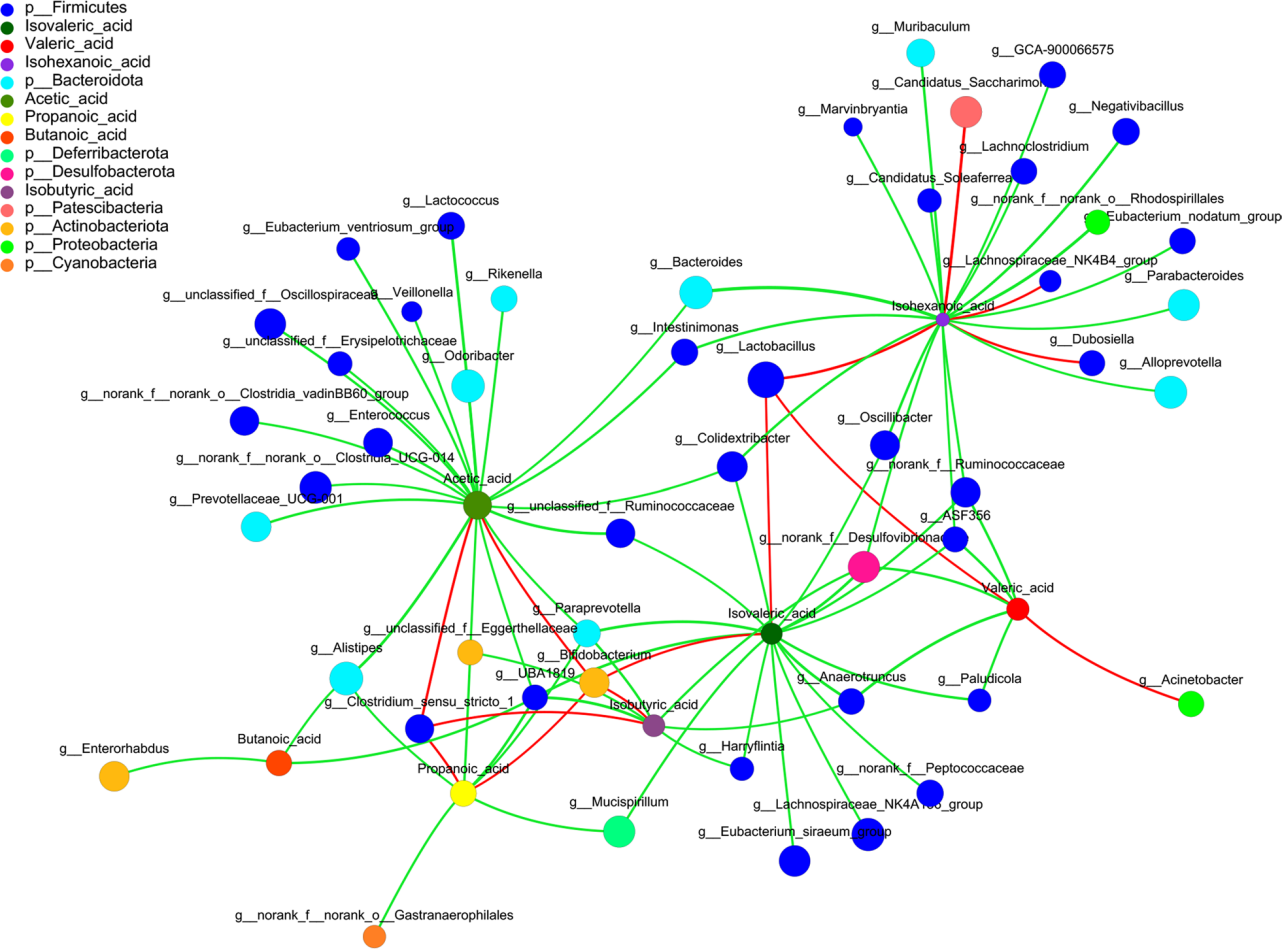
Correlation analysis between short-chain fatty acids (SCFAs) and intestinal flora. The red line represents a positive correlation and the green line represents a negative correlation

We further analyzed the correlation between SCFAs and the intestinal flora (Fig. 5). The positive correlation, represented by the red line, and the green line indicates the negative correlation. Some *Firmicutes*, *Actinobacteriota*, and *Proteobacteria* were positively correlated with SCFAs, such as *Clostridium *sensu stricto* 1* with acetic acid, propionic acid, and isobutyric acid. Positive correlations were also observed between acetic acid, propionic acid, isobutyric acid, and isovaleric acid with *Bifidobacterium;* isovaleric acid, isohexanoic acid, and valeric acid with *Lactobacillus;* and isohexanoic acid with *Lactobacillus, Candidatus Soleaferrea, Lachnospiraceae-NK4B4*, and *Dubosiella*. Other bacteria demonstrated a negative correlation with SCFAs.

## Discussion

Polysaccharides are one of the four basic substances that constitute life; they exist widely in nature, mainly in plant cell walls, animal cell membranes, and microbial cell walls (Xu et al. 2019). Natural polysaccharides have a wide-range of biological effects, including acting as antioxidants (Tang and Huang 2018), immune regulators (Feng et al. 2020), anti-inflammatories (Wang et al. 2019), antibacterial agents (Jridi et al. 2019), hypoglycemic agents (Yang et al. 2019), hypolipidemic agents (Cao et al. 2020), and liver protection agents (Ying et al. 2020), as well as affecting the metabolism of substances and energy in the body to maintain human health (Zhan et al. 2020). However, under normal circumstances, the host lacks enzymes to degrade polysaccharides; therefore, the body cannot directly digest and absorb polysaccharides. Intestinal flora secrete various enzymes to degrade polysaccharides into substances that can be absorbed and utilized by the body, such as SCFAs (Wu et al. 2020), including acetate, propionate, butyrate, and valerate. Our results showed that PsPs upregulated the levels of acetic, propionic, and butyric acid in PsPs-treated mice compared with the ConT group, and these results are consistent with those of previous studies (Wang et al. 2017).

In addition to providing energy for intestinal epithelial cells, SCFAs play an essential role in maintaining water and electrolyte balance, adjusting the balance of intestinal flora, improving bowel function and the resistant microorganisms, anti-inflammatory, preventing obesity, and type 2 diabetes (T2DM) (Fang et al. 2019; Canfora et al. 2019). In addition to being an important energy source for intestinal cells, acetic acid also activates G-protein-coupled receptors, thus activating adipose-insulin signal transduction (Ikuo 2014). Acetic acid, one of the major metabolites of the intestinal tract, not only reduces appetite by directly stimulating the nervous system, but it also prevents obesity-related hyperinsulinemia and hypertriglyceridemia (Ana et al. 2018). Propionic acid participates in immune regulation and reduces high fatty acid levels in the liver and plasma (Sa'ad et al. 2010). Propionic acid also increases the number of enteric-derived regulatory T cells and positively affects the central nervous system by increasing myelin regeneration (Hirschberg et al. 2019). Short-term rectal administration of propionate improved depressive symptoms in chronic unpredictable mild stress (CUMS) model rats (Jianguo et al. 2018). Butyric acid stimulates the expression of fatty acid oxidation genes, thus lowering total cholesterol in the liver (Gail et al. 2017). Butyric acid can also increase the concentration of the central neurotransmitter 5-HT, promote the expression of brain-derived neurotrophic factor (BDNF), and significantly improve depression-like behavior in CUMS model mice (Sun et al. 2016). Our results show that PsPs are fermented to produce different SCFAs, suggesting that the intake of PsPs is beneficial to health.

The intestinal flora is composed of a variety of microorganisms, such as bacteria, fungi, and viruses, that inhabit the gastrointestinal tract of the host. The human gut flora affects the expression of genes, the products of which are involved in the digestion of carbohydrates, proteins, and drugs, providing nutrients to the body (Strozzi and Mogna 2008; Arumugam et al. 2011; Pennisi 2010). We analyzed the gut microbiota profiles of fecal samples through bioinformatics analysis, showing that PsPs can influence the microbiota composition and structure. This is consistent with the results of Gu et al. (2020) showing that polysaccharides extracted from the rhizomes of *Polygonatum* can regulate intestinal microbial structure and composition. *Firmicutes* and *Bacteroidetes* accounted for more than 80% of the total microbiota and were the most dominant flora. The phyla *Firmicutes* and *Bacteriaceae* contain a large number of glycoside hydrolases (Kaoutari et al. 2013) that can aid in the digestion of non-digestible polysaccharides in the gastrointestinal tract, and they are the two most abundant bacteria. *Firmicutes* are considered the main butyrate producer and are difficult to digest in the gut, leading to the degradation of polysaccharides (Kumar et al. 2018). *Lactobacillus* is a typical probiotic among the *Firmicutes*, which regulates intestinal flora and can effectively inhibit intestinal infections (Meizhong et al. 2013). By promoting the colonization of beneficial microorganisms in the intestinal tract, the composition and quantity of SCFAs in the intestinal tract are changed (Maynard et al. 2012), thus resisting the invasion of pathogenic microorganisms and enhancing the function of the intestinal barrier (Guilloteau et al. 2010). Both *Firmicutes* and *Bacteroidetes* can produce butyric acid (Fei et al. 2018), which is produced primarily by *Lactobacillus,* suggesting that there are two ways to promote gut health: by increasing the number of probiotics, and by promoting SCFA synthesis. In this study, *Bacteroidetes* and *Firmicutes* were the two major bacterial phyla in the intestinal tract which could be regulated by PsPs. PsPs significantly changed the relative abundance of *Firmicutes* and *Bacteroidetes*, consistent with previous results (Yang 2021). Our results suggest that PsPs have the same prebiotic effects as the polysaccharides derived from the rhizomes of *Polygonatum* and can promote the production of SCFAs by regulating the composition and structure of intestinal microorganisms. However, rhizome polysaccharides and leaf polysaccharides regulate different intestinal flora, which may be caused by the different sources of polysaccharides resulting in different compositions and structures of polysaccharides. Studies have shown that rhamnose can produce propionic acid by intestinal microbial fermentation (Harris et al. 2021), whereas arabinose can increase the production of acetic acid, propionic acid, and lactic acid (Tiwari et al. 2019). Our results showed that the monosaccharide composition of PsPs mainly consisted of rhamnose, arabinose, and xylose, suggesting that PsPs may be degraded into these monosaccharides by intestinal microorganisms, and then produce SCFAs through fermentation, thus increasing their content.

In conclusion, *Firmicutes*, *Bacteroidetes*, *Campilobacterota*, and *Deferribacterota* were the main bacterial phyla in the intestinal microbiota of control mice. PsPs change the composition and structure of the intestinal microbes, but the diversity of intestinal flora in the PsPs treatment group was not significantly different from that in the ConT group. PsPs increased the relative abundance of *Firmicutes* and decreased the relative abundance of *Bacteroidetes* at the phylum level. Changes in the composition and structure of intestinal microbiota correlated with increased levels of SCFAs, including acetic acid, propionic acid, isobutyric acid, n-butyric acid, and isovaleric acid in PsPs-treated mice. The increase in SCFAs was related to *Clostridium *sensu stricto* 1, Lactobacillus,* and *Dubosiella*. These results indicate that PsPs from the leaves of *Polygonatum* can increase the production of SCFAs by regulating gut microbiota, and that PsPs have positive prebiotic effects and can be used as prebiotics to regulate the intestinal tract. Polysaccharides extracted from leaves of *Polygonatum* have similar biological activities with those of polysaccharides extracted from their rhizomes; thus, the source of polysaccharides is not limited to the rhizomes. This not only reduces plant waste, but makes full use of the resources of *Polygonum*. However, this study had some limitations: PsPs are not a single polysaccharide, and no high and low doses were compared; the mechanism of PsPs on intestinal flora is not clear at present; and the degradation mechanism of PsPs by intestinal flora is also unclear and needs further study.

## Data Availability

All data were uploaded to the National Center for Biotechnology Information database (Accession Number: PRJNA770123).

## References

[CR1] Ana MV, Jens W, Eran S, Tim DS (2018). Role of the gut microbiota in nutrition and health. BMJ.

[CR2] Arumugam M, Raes J, Pelletier E, Paslier DL, Yamada T, Mende DR (2011). Erratum: enterotypes of the human gut microbiome. Nature.

[CR3] Canfora EE, Meex RCR, Venema K, Blaak EE (2019). Gut microbial metabolites in obesity, NAFLD and T2DM. Nat Rev Endocrinol.

[CR4] Cao Y, Zou L, Li W, Song Y, Zhao G, Hu Y (2020). Dietary quinoa (*Chenopodium**quinoa* Willd.) polysaccharides ameliorate high-fat diet-induced hyperlipidemia and modulate gut microbiota. Int J Biol Macromol.

[CR5] Dai J, WuY SW, Chen ZhuS, Yin HR, Wang M, Tang, (2010). Sugar compositional determination of polysaccharides from *Dunaliella salina* by modified RP—HPLC method of precolumn derivatization with 1-phenyl-3-methyl-5-pyrazolone. Carbohyd Polym.

[CR6] Debnath T, Park SR, Kim DH, Jo JE, Lim BO (2013). Antioxidant and anti-inflammatory activity of *Polygonatum**sibiricum* rhizome extracts. Asian Pac J Trop Dis.

[CR7] Fang Q, Hu J, Nie Q, Nie S (2019). Effects of polysaccharides on glycol metabolism based on gut microbiota alteration. Trends Food Sci Technol.

[CR8] Fei J, Luo JT, Zhang XY, Yu XW, Ye JP (2018). The role of short-chain fatty acids in the regulation of human energy metabolism by intestinal flora. Chin J Diabetes.

[CR9] Feng Y, Zhang J, Wen C, Dzah CS, Juliet IC, Duan Y (2020). Recent advances in *Agaricus bisporus* polysaccharides: extraction, purification, physicochemical characterization and bioactivities. Process Biochem.

[CR10] Gail AC, Bryan G, Megan RM, Wei X, Daniella A, Laura EN (2017). Prophylactic tributyrin treatment mitigates chronic-binge ethanol-induced intestinal barrier and liver injury. J Gastroenterol Hepatol.

[CR11] Gou XR, Hui L, Zhang KL, Ke FM, Can S (2020). Diversification of animal gut microbes and NRPS gene clusters in some carnivores, herbivores and omnivores. Biotechnol Biotechnol Equip.

[CR12] Gu W, Wang Y, Zeng L, Dong J, Bi Q, Yang X, Che Y, He S, Yu J (2020). Polysaccharides from Polygonatum kingianum improve glucose and lipid metabolism in rats fed a high fat diet. Biomed Pharmacother.

[CR13] Guilloteau P, Martin L, Eeckhaut V, Ducatelle R, Zabielski R, Immerseel FV (2010). From the gut to the peripheral tissues: the multiple effects of butyrate. Nutr Res Rev.

[CR14] Harris HC, Morrison DJ, Edwards CA (2021). Impact of the source of fermentable carbohydrate on SCFA production by human gut microbiota *in vitro* - a systematic scoping review and secondary analysis. Crit Rev Food Sci Nutr.

[CR15] Herbert D, Phipps PJ, Strange RE (1971). Chemical analysis of microbial cells. Methods Microbiol.

[CR16] Hirschberg S, Gisevius B, Duscha A, Haghikia A (2019). Implications of diet and the gut microbiome in neuroinflammatory and neurodegenerative diseases. Int J Mol Sci.

[CR17] Ikuo K (2014). Host energy regulation via SCFAs receptors, as dietary nutrition sensors, by gut microbiota. Yakugaku Zasshi.

[CR18] Jia N, Qiao H, Zhu W, Zhu M, Meng Q, Lu Q (2019). Antioxidant, immunomodulatory, oxidative stress inhibitory and iron supplementation effect of *Astragalus membranaceus* polysaccharide-iron (III) complex on iron-deficiency anemia mouse model. Int J Biol Macromol.

[CR19] Jianguo L, Luwen H, Cui W, Xueyang J, Xuemei Q, Changxin W (2018). Short term intrarectal administration of sodium propionate induces antidepressant-like effects in rats exposed to chronic unpredictable mild stress. Front Psych.

[CR20] Jridi M, Nasri R, Marzougui Z, Abdelhedi O, Hamdi M, Nasri M (2019). Characterization and assessment of antioxidant and antibacterial activities of sulfated polysaccharides extracted from cuttlefish skin and muscle. Int J Biol Macromol.

[CR21] Juan W, Cheng SL, Dong YL, Yan TX, Yan Z, Hong H (2016). W Constituents from *Polygonatum sibiricum* and their inhibitions on the formation of advanced glycosylation end products. J Asian Nat Prod Res.

[CR22] Jun XY, Shen W, Xi LH, Xiao QH, Yi Z (2015). Hypolipidemic activity and antiatherosclerotic effect of polysaccharide of *Polygonatum sibiricum* in rabbit model and related cellular mechanisms. Evid Based Complement Altern Med.

[CR23] Kaoutari AE, Armougom F, Gordon JI, Raoult D, Henrissat B (2013). The abundance and variety of carbohydrate-active enzymes in the human gut microbiota. Nat Rev Microbiol.

[CR24] Kim H, Kim HW, Yu KW, Suh HJ (2019). Polysaccharides fractionated from enzyme digests of Korean red ginseng water extracts enhance the immunostimulatory activity. Biol Macromol.

[CR25] Kumar G, Kim CS, Jairam V, Baojun X (2018). Causal relationship between diet-induced gut microbiota changes and diabetes: a novel strategy to transplant *Faecalibacterium prausnitzii* in preventing diabetes. Int J Mol Sci.

[CR26] Li SM, Shu XY, Peng XM, Zeng ZL (2020). Extraction and purification of *Polygonum* polysaccharide. Mod Salt Chem Ind.

[CR27] Li DY, Guan H, Yuan ZY (2021). The pharmacological effects of *Polygonati Rhizoma* and its compound prescription in clinical application of traditional chinese medicine. Asia Pac Tradit Med.

[CR28] Liu L, Dong Q, Dong X-t, Fang J-n, Ding K (2007). Structural investigation of two neutral polysaccharides isolated from rhizome of *Polygonatum sibiricum*. Carbohyd Polym.

[CR29] Lv X, Chen D, Yang L, Zhu N, Li J, Zhao J (2016). Comparative studies on the immunoregulatory effects of three polysaccharides using high content imaging system. Int J Biol Macromol.

[CR30] Lv J, Zhang Y, Tian Z, Liu F, Shi Y, Liu Y (2017). *Astragalus* polysaccharides protect against dextran sulfate sodium-induced colitis by inhibiting NF-κB activation. Int J Biol Macromol.

[CR31] Maynard CL, Elson CO, Hatton RD, Weaver CT (2012). Reciprocal interactions of the intestinal microbiota and immune system. Nature.

[CR32] Meizhong H, Haizhen Z, Chong Z, Jiansheng Y, Zhaoxin L (2013). Purification and characterization of *plantaricin 163*, a novel bacteriocin produced by *Lactobacillus plantarum 163* isolated from traditional Chinese fermented vegetables. J Agric Food Chem.

[CR33] Mi-Jeong A, Young KC, Kee-Dong Y, Youl RM, Hye CJ, Young-Won C (2006). Steroidal saponins from the rhizomes of *Polygonatum sibiricum*. J Nat Prod.

[CR34] Pennisi E (2010). Body’s hardworking microbes get some overdue respect. Science.

[CR35] Sa'ad HA, Maikel PP, Roelofsen H, Roel JV, Roel V (2010). Biological effects of propionic acid in humans; metabolism, potential applications and underlying mechanisms. Biochim Biophys Acta.

[CR36] Sajadimajd S, Momtaz S, Haratipour P, El-Senduny FF, Panah AI, Navabi J, Soheilikhah Z, Farzaei MH, Rahimi R (2019). Molecular mechanisms underlying cancer preventive and therapeutic potential of algal polysaccharides. Curr Pharm Des.

[CR37] Senthilkumar K, Manivasagan P, Venkatesan J, Kim SK (2013). Brown seaweed fucoidan: biological activity and apoptosis, growth signaling mechanism in cancer. Biol Macromol.

[CR38] Shalaby ASG, Ragab TIM, Mehany ABM, Helal MMI, Helmy WA (2018). Antitumor and prebiotic activities of novel sulfated acidic polysaccharide from *Ginseng*. Biocatal Agric Biotechnol.

[CR39] Shu X, Lv J, Chen D, Chen Y (2012). Herald. Biochem Bioinform.

[CR40] Strozzi GP, Mogna L (2008). Quantification of folic acid in human feces after administration of *Bifidobacterium* probiotic strains. J Clin Gastroenterol.

[CR41] Sun LR, Li X, Wang SX (2005). Two new alkaloids from the rhizome of *Polygonatum sibiricum*. J Asian Nat Prod Res.

[CR42] Sun J, Wang F, Hong G, Pang M, Xu H, Li H (2016). Antidepressant-like effects of sodium butyrate and its possible mechanisms of action in mice exposed to chronic unpredictable mild stress. Neurosci Lett.

[CR43] Tang Q, Huang G (2018). Preparation and antioxidant activities of *Cuaurbit* polysaccharide. Int J Biol Macromol.

[CR44] Tiwari UP, Singh AK, Jha R (2019). Fermentation characteristics of resistant starch, arabinoxylan, and ß-glucan and their effects on the gut microbial ecology of pigs: a review. Anim Nutr.

[CR45] Wang Y, Fei Y, Liu L, Xiao Y, Pang Y, Kang J, Wang Z (2018). *Polygonatum**odoratum* polysaccharides modulate gut microbiota and mitigate experimentally induced obesity in rats. Int J Mol Sci.

[CR46] Wang Y, Ji X, Yan M, Chen X, Kang M, Teng L (2019). Protective effect and mechanism of polysaccharide from *Dictyophora indusiata* on dextran sodium sulfate-induced colitis in C57BL/6 mice. Int J Biol Macromol.

[CR47] Wang H, Yuan D P, Zeng C H (2017) Advance sinpharmacological effects and clinical application of Polygonatum. Hubei Univ Natl·Med. 2: 58–60

[CR48] Wang YF (2017) Study on the effect of *Polygonatum* polysaccharide on lipid metabolism disorder in rats. Yunnan University of Traditional Chinese Medicine

[CR49] Wu K, Fan J, Huang X, Wu X, Guo C (2018). Hepatoprotective effects exerted by *Poria Cocos* polysaccharides against acetaminophen-induced liver injury in mice. Int J Biol Macromol.

[CR50] Wu YY, Xie RZ, Lin Y, Zhou YR, Zhou FM (2020). Research progress in the interaction between Polysaccharides from traditional chinese medicine and intestinal microflora. J Pract Chin Med.

[CR51] Xiao WC, Shi YW, Hui C, Hong G, Yu JL, Fang XX (2018). A review: the bioactivities and pharmacological applications of *Polygonatum sibiricum* polysaccharides. Molecules (basel, Switzerland).

[CR52] Xu Y, Wu YJ, Sun PI, Zhang FM, Linhardt RJ, Zhang AQ (2019). Chemically modified polysaccharides: Synthesis, characterization, structure activity relationships of action. Int J Biol Macromol.

[CR53] Yang M (2021). Digestibility of *Polygonum* polysaccharide in vitro and its regulation of intestinal microflora in type II diabetic mice. Mod Food Sci Technol.

[CR54] Yang C, Feng Q, Liao H, Yu X, Liu Y, Wang D (2019). Anti-diabetic nephropathy activities of polysaccharides obtained from *Termitornyces albuminosus* via regulation of NF-κB Signaling in db/db mice. Int J Mol Sci.

[CR55] Yang CJ, Yan P, Luo Y, Mu YH, Liu MK, Gao P (2020). Optimization of water extraction process for polysaccharides from leaves of polygonatum by response surface methodology. J Sichuan Univ Nat Sci Edi.

[CR56] Yang M, Meng F, Gu W, Fu L, Zhang F, Li F, Tao Y, Zhang Z, Wang X, Yang X, Li J, Yu J (2021). Influence of polysaccharides from *Polygonatum kingianum* on short-chain fatty acid production and quorum sensing in *Lactobacillus faecis*. Front Microbiol.

[CR57] Ying Y, Jing J, Liuqing D, Junsong L, Lihong H, Hongzhi Q (2020). Resource, chemical structure and activity of natural polysaccharides against alcoholic liver damages. Carbohydr Polym.

[CR58] Zeng GF, Zhang ZY, Lu L, Xiao DQ, Xiong CX, Zhao YX (2011). Protective effects of *Polygonatum sibiricum* polysaccharide on ovariectomy-induced bone loss in rats. J Ethnopharmacol.

[CR59] Zhan Y, An X, Wang S, Sun M, Zhou H (2020). *Basil* polysaccharides: a review on extraction, bioactivities and pharmacological applications. Bioorg Med Chem.

[CR60] Zhao P, Zhao C, Li X, Gao Q, Huang L, Xiao P (2018). The genus *Polygonatum*: a review of ethnopharmacology, phytochemistry and pharmacology. J Ethnopharmacol.

